# Protocol of the Comparison of Intravesical Therapy and Surgery as Treatment Options (CISTO) study: a pragmatic, prospective multicenter observational cohort study of recurrent high-grade non-muscle invasive bladder cancer

**DOI:** 10.1186/s12885-023-11605-8

**Published:** 2023-11-18

**Authors:** John L. Gore, Erika M. Wolff, Bryan A. Comstock, Kristin M. Follmer, Michael G. Nash, Anirban Basu, Stephanie Chisolm, Douglas B. MacLean, Jenney R. Lee, Yair Lotan, Sima P. Porten, Gary D. Steinberg, Sam S. Chang, Scott M. Gilbert, Larry G. Kessler, Angela B. Smith, CISTO Collaborative, Patrick J. Heagerty, On H. Ho, Sung Min Kim, Solange Mecham, Christopher Nefcy, Jeffrey C. Bassett, Trinity J. Bivalacqua, Karim Chamie, David Y. T. Chen, Siamak Daneshmand, Rian Dickstein, Adam J. Gadzinski, Thomas J. Guzzo, Ashish M. Kamat, Max R. Kates, Janet B. Kukreja, Brian R. Lane, Eugene K. Lee, Liam C. Macleod, Ahmed M. Mansour, Viraj A. Master, Parth K. Modi, Jeffrey S. Montgomery, David S. Morris, Matthew Mossanen, Kenneth G. Nepple, Jeffrey W. Nix, Brock B. O’Neil, Sanjay Patel, Charles C. Peyton, Kamal S. Pohar, Chad R. Ritch, Alex Sankin, Kristen R. Scarpato, Neal D. Shore, Mark D. Tyson, Mary E. Westerman, Solomon L. Woldu, Stephanie Chisolm, Jonathan L. Wright, Fred Almeida, Mary Beth Ballard Murray, Nancy Lindsey, Robert Lipman, Rick M. Oliver, Lori A. Roscoe, Karen Sachse, James W. F. Catto, Tracy M. Downs, Tullika Garg, Ewan A. Gibb, Jennifer L. Malin, Jennifer M. Taylor

**Affiliations:** 1 Department of Urology, University of Washington, Seattle, WA USA; 2 Department of Biostatistics, University of Washington, Seattle, WA USA; 3 Departments of Pharmacy, Health Services, and Economics, University of Washington, Seattle, WA USA; 4 Bladder Cancer Advocacy Network, Bethesda, MD USA; 5 CISTO Advocate Advisory Board, Carnation, WA USA; 6 Department of Urology, University of Texas Southwestern Medical Center, Dallas, TX USA; 7 Department of Urology, UCSF School of Medicine, San Francisco, CA USA; 8 Department of Urology, Rush University Medical Center, Chicago, IL USA; 9 Department of Urology, Vanderbilt University Medical Center, Nashville, TN USA; 10 Department of Genitourinary Oncology, H. Lee Moffit Cancer Center and Research Institute, Tampa, FL USA; 11 Department of Health Systems and Population Health, School of Public Health, University of Washington, Seattle, WA USA; 12 Department of Urology, School of Medicine, University of North Carolina at Chapel Hill, Chapel Hill, NC USA

**Keywords:** Non-muscle invasive bladder cancer, Pragmatic trial, Radical cystectomy, Administration, intravesical, Patient-centered care, Quality of life, Observational study

## Abstract

**Background:**

Bladder cancer poses a significant public health burden, with high recurrence and progression rates in patients with non-muscle-invasive bladder cancer (NMIBC). Current treatment options include bladder-sparing therapies (BST) and radical cystectomy, both with associated risks and benefits. However, evidence supporting optimal management decisions for patients with recurrent high-grade NMIBC remains limited, leading to uncertainty for patients and clinicians. The CISTO (Comparison of Intravesical Therapy and Surgery as Treatment Options) Study aims to address this critical knowledge gap by comparing outcomes between patients undergoing BST and radical cystectomy.

**Methods:**

The CISTO Study is a pragmatic, prospective observational cohort trial across 36 academic and community urology practices in the US. The study will enroll 572 patients with a diagnosis of recurrent high-grade NMIBC who select management with either BST or radical cystectomy. The primary outcome is health-related quality of life (QOL) at 12 months as measured with the EORTC-QLQ-C30. Secondary outcomes include bladder cancer-specific QOL, progression-free survival, cancer-specific survival, and financial toxicity. The study will also assess patient preferences for treatment outcomes. Statistical analyses will employ targeted maximum likelihood estimation (TMLE) to address treatment selection bias and confounding by indication.

**Discussion:**

The CISTO Study is powered to detect clinically important differences in QOL and cancer-specific survival between the two treatment approaches. By including a diverse patient population, the study also aims to assess outcomes across the following patient characteristics: age, gender, race, burden of comorbid health conditions, cancer severity, caregiver status, social determinants of health, and rurality. Treatment outcomes may also vary by patient preferences, health literacy, and baseline QOL. The CISTO Study will fill a crucial evidence gap in the management of recurrent high-grade NMIBC, providing evidence-based guidance for patients and clinicians in choosing between BST and radical cystectomy. The CISTO study will provide an evidence-based approach to identifying the right treatment for the right patient at the right time in the challenging clinical setting of recurrent high-grade NMIBC.

**Trial registration:**

ClinicalTrials.gov, NCT03933826. Registered on May 1, 2019.

**Supplementary Information:**

The online version contains supplementary material available at 10.1186/s12885-023-11605-8.

## Background

Bladder cancer is the sixth most common cancer in the United States and affects all genders.^1^ Each year, more than 80,000 people residing in the United States are diagnosed with bladder cancer. Most bladder cancer patients (74%) present with non-muscle-invasive bladder cancer (NMIBC), where the cancer is limited to the lining or support layer of the bladder [1]. NMIBC has the oldest median age at diagnosis among cancer types, intensive surveillance requirements, [2, 3] high recurrence and progression rates (up to 80% and 44%, respectively), [4] and one of the greatest lifetime treatment costs of all cancers [5]. Most high-grade NMIBC is treated initially with endoscopic resection and intravesical immunotherapy with bladder instillations of bacillus Calmette-Guérin (BCG) [1, 2, 6]. However, 24%–61% of patients will have their cancers recur within 12 months of BCG treatment, and these individuals have limited treatment options [7]. National guidelines recommend consideration between two alternatives: bladder-sparing therapies (BSTs, with significant risk of cancer recurrence and/or progression) or radical cystectomy (a life-altering bladder removal surgery with substantial short-term morbidity and mortality) [2, 3]. Selecting between these options involves weighing the risk of progression of bladder cancer and loss of a window of potential cure versus the risk of morbidity and lifelong impact on daily life with bladder removal. However, patients, their caregivers, and clinicians must make this complex treatment decision based on limited evidence [8–10]. Thus, there is a critical need for high-quality research in recurrent high-grade NMIBC across the full spectrum of outcomes to inform treatment decision-making.

We conducted an Agency for Healthcare Research and Quality (AHRQ)-funded evidence review of the comparative effectiveness of numerous intravesical treatments and identified a paucity of evidence for the effectiveness of management strategies for patients with recurrent high-grade NMIBC [11]. Thus, contemporary research provides limited evidence to guide patient and clinician decision-making. Through a Patient-Centered Outcomes Research Institute (PCORI) Engagement Award (Contract 1089), we partnered with the Bladder Cancer Advocacy Network (BCAN) to organize a 1,300-member Patient Survey Network for patient-centered research prioritization [12]. The top prioritized questions among NMIBC patients were: “How can patients and providers make decisions about the need for radical cystectomy and what is the best timing?” and “What are the best treatments for patients whose cancer returns or worsens after BCG treatment?” These questions are similar to those prioritized by patients and providers in Europe as well [13]. Thus, clarifying the role of BST versus radical cystectomy is a predominant concern among NMIBC patients. Similarly, in early engagement work with clinicians, they noted that management of recurrent high-grade NMIBC was a tremendous challenge since there had been no large prospective studies conducted that directly compared the impact of these two strategies on clinical or patient-reported outcomes (PROs) [14].

To address this clinical and patient-centered gap, we designed the Comparison of Intravesical Therapy and Surgery as Treatment Options (CISTO) Study in partnership with patients and other stakeholders engaged in NMIBC care, including clinicians and policymakers, and received funding from PCORI to support the CISTO Study as a 36-site pragmatic trial (PI: Gore, NCT03933826) [14, 15]. The CISTO Study includes outcomes that are important to patients, caregivers, and clinicians (i.e., quality of life, cancer progression, mortality, financial toxicity). The primary outcome of the CISTO Study is to compare health-related quality of life (QOL) at 12 months. Secondary outcomes include progression-free, cancer-specific, and overall survival as well as the PROs of bladder-cancer specific QOL, decision regret, health state utilities, patient preferences, and financial toxicity. The CISTO Study will include 572 patients with a minimum of 12 months of follow-up. Broad eligibility criteria ensure that results will be generalizable to patients seen in urology clinics across the United States. The CISTO study has the potential to serve as the foundation for addressing critical questions relevant to the optimal patient-centered management of recurrent high-grade NMIBC.

## Methods/design

### Aims

The primary objective of the CISTO Study is to compare patient-reported and patient-centered clinical outcomes between patients undergoing radical cystectomy and those receiving BST for recurrent high-grade NMIBC. We hypothesize that: 1) patients undergoing radical cystectomy will have worse health-related QOL at 12 months compared with patients who choose BST; 2) 12-month disease-free survival and metastasis-free survival will be better among radical cystectomy patients than among patients who choose BST; and 3) treatment with BST will result in better QOL within important subgroups, such as those aged 75 years or older, women, and patients with multiple comorbid health conditions. A secondary objective of the study is to characterize the heterogeneity of treatments received and corresponding patient preferences.

### Study design

The CISTO Study is a pragmatic, prospective observational cohort study of patients with recurrent high-grade NMIBC who have selected management with BST or radical cystectomy. Since radical cystectomy is selected less frequently than BST, the study design includes a cap of two enrollments into the BST arm for every one enrollment into the cystectomy arm at each site. This block recruitment strategy ensures adequate enrollment into the BST arm and limits unbalanced accrual by sites. A prospective observational cohort study design was chosen for the CISTO Study in response to critical input from the BCAN Patient Survey Network, as only 11% of respondents reported being willing to consent to randomization for a study of BST versus radical cystectomy [14].

**Fig. 1:**
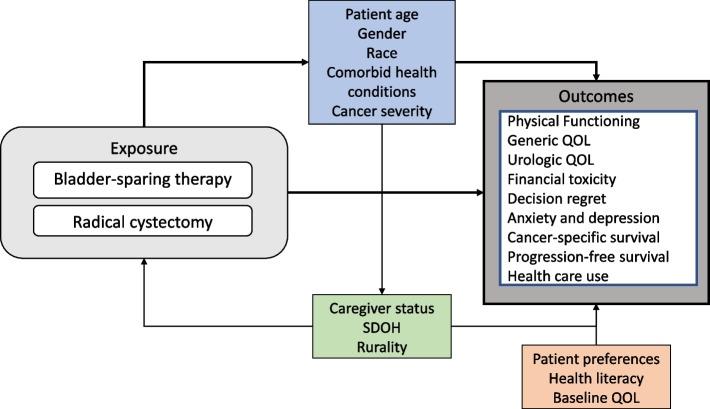
Conceptual model for the CISTO Study. The CISTO Study design is informed by a conceptual model of the relationship between bladder-sparing therapies (BST) and radical cystectomy and the planned patient-centered outcomes. Since the decision to select BST or radical cystectomy may relate to patient factors, we aim to assess patient-reported outcomes (PROs) and clinical outcomes across the following patient characteristics: age, gender, race, burden of comorbid health conditions, cancer severity, caregiver status, social determinants of health (SDH), and rurality. Treatment outcomes may also vary by patient preferences, health literacy, and baseline quality of life (QOL)

The CISTO Study is informed by a conceptual model of the relationship between BST and radical cystectomy and patient-centered outcomes (Fig. 1). As such, we will assess PRO and oncologic outcomes across the following patient characteristics: age, gender, race, burden of comorbid health conditions, and cancer severity. Women comprise 20% of patients with bladder cancer and present at more advanced stages [16]. Black patients present with more advanced bladder cancer [16]. Those over 75 years old and those with multiple comorbid conditions are at increased risk for complications after radical cystectomy [17, 18]. Clinical severity can be represented by tumor stage or the presence of variant histology that can exhibit aggressive behavior. Lastly, the decision to select BST or radical cystectomy may relate to patient factors such as their caregiver status, social determinants of health (SDH), rurality, and the costs of treatment.

**Fig. 2:**
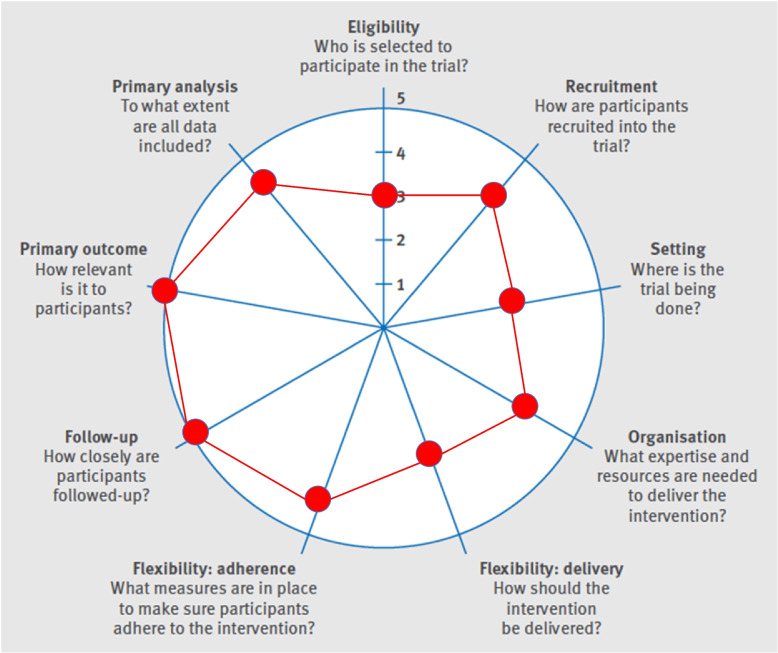
Pragmatism of the CISTO Study based on PRECIS-2 criteria. The level of pragmatism for each criteria was assessed as follows: Eligibility: similarity of patients to usual care; Recruitment: via usual care; Setting: 36 clinical sites, including a mixture of community and academic hospitals, which mimics where results will be applied; Organization: no clinician training required, minimal research staff training; Flexibility of delivery: mimics usual practice; Flexibility of adherence: Enforcement of compliance; Follow-up: clinical outcomes data collected using a web-based research portal, including uploading of clinical notes and other documents from the electronic health record (EHR), also multi-modal survey options; Primary outcome: patient-centered, not disease-centered, and important to all stakeholders; and Primary analysis: data included for all participants utilizing an intention-to-treat (ITT) analysis. *PRECIS-2* PRagmatic-Explanatory Continuum Indicatory Summary 2

The CISTO Study protocol was developed through multistakeholder involvement as described previously, [14] applying the PRagmatic-Explanatory Continuum Indicatory Summary 2 (PRECIS-2) tool to maximize the applicability of the study results (Fig. 2), [19] and in accordance with the Standard Protocol Items: Recommendations for Interventional Trials (SPIRIT) Statement [20, 21].

### Study setting

The study setting is academic and community urology practices in the United States. A total of 36 clinical sites were selected based on geography, patient volume, patient demographics, research infrastructure, and previous experience with clinical trials.

### Participants

The CISTO Study will include 572 patients with a diagnosis of recurrent high-grade NMIBC who chose either cystectomy or BST as their treatment option. Participants must have attempted or received induction BCG at some point and have received their last treatment for NMIBC within the previous 12 months (Table 1). Key exclusion criteria are a history of muscle-invasive bladder cancer or upper urinary tract urothelial cancer, not being a candidate for either treatment arm as determined by the treating physician, or planning to participate in a Phase I or Phase II interventional clinical trial for NMIBC. The broad inclusion criteria and limited exclusion criteria for this pragmatic study allow for the inclusion of patients with additional categories of recurrent high-grade NMIBC who are typically excluded from clinical trials (Table 2) and will improve the generalizability of the study results.

**Table 1:** The CISTO Study inclusion and exclusion criteria

Inclusion Criteria	Exclusion Criteria
1. Adult 18 years of age or older; and 2. Presenting with high-grade NMIBC established by anatomic pathology as tumor stage classification Tis, Ta, or T1, and with: a. Pathology documentation from any hospital/clinic/medical center, and b. More than 50% urothelial carcinoma component in the specimen; and 3. History of high-grade NMIBC established by anatomic pathology as tumor stage classification Tis, Ta, or T1; and 4. Attempted or received induction BCG (at least 3 out of 6 instillations) at any point in time; and 5. In the previous 12 months, received at least one instillation of any intravesical agent (induction or maintenance) or one administration of systemic therapy for NMIBC treatment	1. Any plasmacytoid or small cell (neuroendocrine) component in the pathology (past or current presentation); 2. Previous history of cystectomy or radiation therapy for bladder cancer; 3. Previous history of muscle-invasive bladder cancer or metastatic bladder cancer; 4. Any history of upper tract urothelial carcinoma; 5. Incarcerated in a detention facility or in police custody (patients wearing a monitoring device can be enrolled) at baseline/screening; 6. Contraindication to radical cystectomy (e.g., ASA classification of 4, cancer does not warrant consideration of cystectomy); 7. Contraindication to medical therapy (i.e., intolerant of all medical therapies); 8. Unable to provide written informed consent in English; 9. Unable to be contacted for research surveys; 10. Planning to participate in a Phase I or Phase II interventional clinical trial for NMIBC (unless in the control/comparator arm of a Phase II trial) or any blinded interventional trial for NMIBC

*NMIBC* Non-muscle invasive bladder cancer, *BCG* Bacillus Calmette-Guérin, *ASA* American Society of Anesthesiologists

**Table 2:** Categories of recurrent high-grade NMIBC included in the CISTO study

Category	Definition
1a. BCG Unresponsive	Persistent or recurrent high-grade NMIBC (stage Tis, Ta, or T1) determined at 6-month evaluation. Patient must have had 5 out of 6 induction BCG instillations and 2 out of 3 maintenance BCG instillations
1b. BCG Unresponsive^a^	Any stage progression at 3-month evaluation despite 5 out of 6 induction BCG instillations
2. Relapsing	Recurrent high-grade NMIBC more than 6 months after diagnosis. Patient must have had at least 3 out of 6 induction BCG instillations and had a maintenance BCG instillation within the last 6 months
3. No recent BCG^a^	Had at least 3 of 6 induction BCG instillations at any point in time. Must have had at least one instillation of some type of intravesical agent (induction or maintenance; postop instillation does not apply) or one administration of systemic therapy approved for NMIBC treatment in last 12 months

*NMIBC* Non-muscle invasive bladder cancer, *BCG* Bacillus Calmette-Guérin

^a^Typically excluded from clinical trials

### Recruitment and retention

**Fig. 3:**
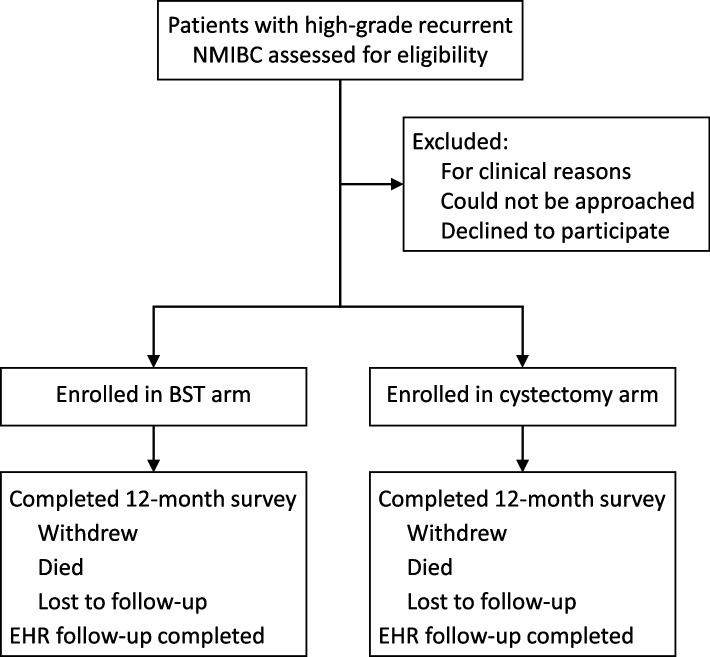
CISTO Study overview. Patients with high-grade recurrent non-muscle invasive bladder cancer (NMIBC) seen at urology clinics from the 36 participating CISTO Study sites are assessed for eligibility. Eligible patients are approached for participation and complete an informed consent process. Participants are enrolled in either the bladder-sparing therapy (BST) or cystectomy arm based on their individual treatment decision. All participants are followed for at least 12 months, including by survey and abstraction from the electronic health record (EHR)

Patients being seen for recurrent high-grade NMIBC at urology clinics are approached for participation (Fig. 3). Patients are identified through review of clinic and operating room schedules. Research coordinators screen for eligibility, confirm eligibility with the provider, approach the patient, and complete an informed consent process. As described previously, [14] a short introductory video that was developed with stakeholders is available to facilitate the recruitment process. Documentation of consent is supported through a variety of modalities (paper and electronic) to provide flexibility for research staff in their recruitment strategies. Participants complete a baseline survey prior to starting treatment, can fill out baseline and follow-up surveys either on paper or electronically, and receive a stipend for each survey completed. Follow-up survey timepoints include 3, 6, 9, and 12 months for all participants, with additional surveys every 6 months thereafter up to 48 months depending on study enrollment date. Follow-up surveys are administered centrally by the clinical coordinating center at the University of Washington, including automated email surveys, mailed paper surveys, text message links to access surveys, and reminder phone calls. Research coordinators abstract data from the electronic health record (EHR) at the time of enrollment and annually thereafter. If a patient requests to withdraw from the study, no additional surveys are sent and no data from after that date will be abstracted from the EHR.

### Interventions

Patients are enrolled in either of two arms, based on the patient’s individual treatment decision: participants undergoing radical cystectomy (any surgical approach including open or robotic surgery) and those receiving BST (additional intravesical therapy, including BCG, or intravenous immunotherapy) for recurrent high-grade NMIBC.

### Comparisons

The primary outcome is patient-reported physical QOL measured with the European Organisation for Research and Treatment of Cancer Core Quality of Life (EORTC QLQ-C30) physical functioning domain at 12 months (Table 3) [22]. The EORTC QLQ-C30 has been used to assess health-related QOL in bladder cancer patients and has been extensively validated [23]. Secondary PROs include generic QOL with the EORTC QLQ-C30 [22] and EuroQoL EQ-5D-5L, [24] self-reported urinary and sexual function measured with the Bladder Cancer Index (BCI), [25] PROMIS-29 (Patient Reported Outcome Measurement Information System) anxiety and depression domains, [26] decision regret, [27, 28] financial distress, [29] and NMIBC treatment preferences at 12 months. The 5-level EQ-5D (EQ-5D-5L) is a generic health-related QOL instrument that assesses health status not specific to a health condition or patient group [24]. The BCI assesses symptom magnitude and symptom impairment in the bladder cancer-specific domains of urinary health, bowel health, and sexual health [25]. Survey items are gender neutral and agnostic to cystectomy history, thus the BCI may be answered by all patients who have retained their bladders or who have undergone radical cystectomy. PROMIS-29 (v2.1) is a collection of 4-item short forms assessing numerous domains, of which we will include anxiety and depression [26]. PROMIS-29 has been extensively validated with population norms for common health conditions and has been used previously in cancer populations [26, 30, 31]. We will assess decision regret with questions modified from a separate two-item regret scale [27, 28]. Financial distress will be measured with the Comprehensive Score for Financial Toxicity (COST), a validated assessment of financial distress that patients experience as a result of their cancer [29]. We will construct utilities for the treatment outcomes participants may experience using time tradeoff (TTO) questions, in which patients indicate how many years of life they would be willing to “trade off” in order to experience perfect health instead of (1) their current health or (2) projected negative health outcomes of retained bladder and non-metastasized NMIBC, removed bladder and non-metastasized NMIBC, and metastasized bladder cancer. Secondary clinical endpoints include cancer-specific survival and progression to muscle-invasive or metastatic bladder cancer at 12 months. Table 3 details the primary and secondary outcome assessments.

**Table 3:** The CISTO Study primary and secondary outcomes

**Name of Outcome**	**Specific Measure**	**Timepoints (months)**^c^
Physical functioning	EORTC QLQ-C30 [22] physical functioning domain	3, 6, 9, 12^a^
Health-related QOL	EORTC QLQ-C30 [22]	3, 6, 9, 12^b^
Bladder cancer-specific QOL	Bladder Cancer Index [25]	3, 6, 9, 12^b^
Financial toxicity	Comprehensive Score for Financial Toxicity (COST) measure	12^b^
Decision regret	Decision regret [27, 28]	12^b^
Anxiety & depression	PROMIS-29 anxiety & depression domains [26]	3, 6, 9, 12^b^
Generic QOL/Global utility	EQ-5D-5L [24]	12^b^
Cancer-specific survival	EHR abstraction for mortality events and cause of death	12^b^
Progression-free survival	EHR abstraction for progression to muscle-invasive or radiographic evidence of metastatic bladder cancer	12^b^
Health care utilization	Extra clinic visits, ED visits, home health care	12^b^
Health state preferences	Time tradeoff (TTO) questions	12^b^

*QOL* Quality of life, *EORTC QLQ-C30* European Organisation for Research and Treatment of Cancer Core Quality of Life, *PROMIS* Patient Reported Outcome Measurement Information System, *EHR* Electronic health record, *ED* Emergency Department

^a^Indicates primary outcome

^b^Indicates secondary outcomes

^c^This study will allow for the collection of 12 months of follow up for all participants. Additional surveys are administered every 6 months thereafter up to 48 months depending on study enrollment date

### Data management and statistical analysis

#### Data management

All study data is managed in REDCap to facilitate data collection and monitoring. The quality of data is maintained with training and using standardized data dictionaries. Missing data or erroneous data are identified in automated nightly reports made available to staff at study sites to address. Important data elements are prospectively monitored to examine patterns or reasons for “missingness.” In accordance with the PCORI Policy for Data Management and Data Sharing, all data and metadata generated from the CISTO Study will be deposited in a PCORI-designated data repository within one year after completion of the study and available for third-party requests.

#### Data safety and monitoring plan

The CISTO Study adheres to a Data Safety Monitoring Plan. Given the minimal risk nature of this observational cohort study, monitoring is conducted by the study’s Executive Committee. The Executive Committee reviews data and safety events and procedures and determine recommendations for these events and procedures as appropriate, including identifying, reviewing, and reporting adverse events (AEs) and serious adverse events (SAEs) and unanticipated problems to the applicable Institutional Review Boards (IRBs) or other monitoring bodies. SAEs are defined as 1) death during the study period; 2) a life-threatening event related to the treatment or significant disability/incapacity related to the treatment; 3) inpatient hospitalization (other than for cystectomy); and 4) prolonged hospitalization following cystectomy (14 days or more). The number of adverse events and related unexpected SAEs will be summarized by arm, by grade, and by the National Cancer Institute’s Common Terminology Criteria for Adverse Events (CTCAE) system organ class. In addition, for each toxicity, the proportion of affected participants overall and by arm will be summarized by the maximum CTCAE grade experienced.

#### Statistical analysis

We will use descriptive statistics to characterize the treatments received, PROs, and clinical outcomes. Prior to statistical analysis, we will first compare continuous demographic and clinical characteristics by treatment group with Wilcoxon rank-sum tests to protect against violations of normality assumptions. *P*-values from Exact Conditional Tests, such as Fisher's exact test and its multi-degree of freedom extensions, will be used to compare categorical data.

To address treatment selection bias and potential confounding by indication in the comparison of BST vs. radical cystectomy, we will utilize targeted maximum likelihood estimation (TMLE) as the primary analytic approach for causal effect estimation using observational data [32–34]. Unlike approaches that focus on creating carefully matched treatment and control groups, TMLE allows for the inclusion of all patient participants and their reported outcome measures [32–34]. TMLE first uses covariate-adjusted regression models to generate an initial estimate of the treatment effect of radical cystectomy through the creation of *potential outcomes*: two predicted outcomes for each individual patient participant, under the hypothetical assumption that they had been treated with either radical cystectomy or intravesical therapy. Next, as with a propensity score approach, we will estimate the probability of treatment with radical cystectomy using either logistic regression, machine learning (LASSO, regression trees, etc.) or super learning approaches. Finally, we will use the probabilities obtained in the second step to update the initial estimate of each patient participant’s pair of potential outcomes and the TMLE estimate is interpreted as the causal difference in QOL outcomes if all patients had been treated with chemoradiation versus having been treated with radical cystectomy [35]. As a secondary sensitivity analysis, we will also evaluate these data using propensity score and G-computation methods.

Patient data will be analyzed according to an intention-to-treat (ITT) framework for the primary analysis, where the treatment arm is decided upon at enrollment regardless of whether or not that treatment was initiated. Longitudinal trajectories of the effect of treatment on patient QOL over time will be modeled and characterized using standard linear mixed effects models (primary analysis) or generalized estimating equations (GEE, secondary analysis). Because each patient has a pair of potential outcomes under each treatment, we will include covariates in the TMLE and longitudinal data models (Supplemental Table 1) to provide treatment effect estimates for important subgroups of interest stratified by age, gender, race, burden of comorbid health conditions, and cancer severity. To test the hypothesis that cancer-specific survival will be better in patients undergoing radical cystectomy, we will use a superiority testing framework. For assessment of time-to-event outcomes we will use inverse probability weighted survival models to estimate relative risks. Since time-to-event outcomes (cancer-specific survival and progression-free survival) will be highly correlated with one another, two-sided 95% confidence intervals will be used for inference without adjustment for multiple survival endpoints.

As a part of treatment effect estimation, the TMLE algorithm provides an integrated approach to incorporating uncertainty that arises due to missing longitudinal data. The mean outcome conditional on observing the outcome may be a biased estimate when missingness is informative. TMLE can reduce this bias when missingness is a function of measured baseline demographic and clinical characteristics. In the TMLE algorithm, we will include of a matrix of missing data probabilities conditioned on baseline demographic and clinical characteristics. The missing data conditional probabilities are then incorporated into the TMLE estimation procedure during the third step of updating potential outcomes. As a sensitivity analysis, for patients that die we will impute the floor of each QOL instrument for all subsequent time points to provide death-adjusted treatment effect estimates.

#### Power calculation

With recruitment of 572 participants, this study has > 0.80 statistical power to detect small differences in QOL between treatment approaches (Cohen’s d = 0.24, or 5.5 points on the physical function scale of EORTC-QLQ-C30) [36]. Power analyses conservatively allowed for 10% missing data and assumed a correlation between repeated QOL measurements of 0.3. Assuming a similar balance of treatments within subgroups, this study also has > 0.80 power to detect moderate but clinically important treatment effects (Cohen’s d = 0.43, or 9.9 points on the physical function scale of EORTC-QLQ-C30) within subgroups as small as 30% of the study cohort. Anticipated subgroups of this size include patients aged 75 years or older, women, and patients with multiple comorbid health conditions. For the secondary outcome of bladder cancer-specific mortality, data from a review combining multiple trials demonstrated an anticipated 1-year bladder cancer-specific mortality rate of 4.8% when treated with medical management [37]. Assuming a 4.8% mortality rate in the medical management arm, this study has power to detect differences in 1-year bladder cancer-specific mortality rates of ≤ 1% in the radical cystectomy arm (power > 80%).

## Discussion

Recurrent high-grade NMIBC is a public health burden, with limited evidence to inform the complex decision confronted by patients and clinicians as they consider BST and radical cystectomy. This complex decision-making engages patients and their caregivers, who may be impacted by the urinary, sexual, and bowel dysfunctions that can occur with NMIBC treatment. Optimal decision-making would include evidence-based selection of treatment that optimizes cancer-specific and QOL outcomes consistent with the patient’s values and preferences. The CISTO Study aims to fill the current knowledge gaps regarding the comparative effectiveness and harms of management options for recurrent high-grade NMIBC to inform these critically important decisions. By including a diverse patient population, the study also aims to assess outcomes across the following patient characteristics: age, gender, race, burden of comorbid health conditions, cancer severity, caregiver status, social determinants of health, and rurality. Treatment outcomes may also vary by patient preferences, health literacy, and baseline QOL. The results of the CISTO Study are anticipated to help identify which patients for whom radical cystectomy represents the best option, and similarly which patients are best managed with BST.

The patient-centered and pragmatic CISTO Study has numerous strengths. The primary question this study will address was prioritized by patients, the design of the study was conducted in partnership with patients and clinicians, and the primary and secondary outcomes were selected based on patient and clinician input. The broad inclusion criteria and pragmatic design of the CISTO Study will improve the generalizability of the study results. The experiences of patients will be assessed with validated PROs, and all statistical analyses will apply rigorous methods and sensitivity analyses. Since treatment selection bias is an inherent part of any observational cohort study, we will utilize TMLE as our primary analytic strategy. TMLE is an innovative, state-of-the-art analytic method for causal effect estimation using observational data. Unlike general propensity score-based or G-computation approaches, TMLE is doubly robust to misspecification arising from an omitted confounding variable in the models for the exposure (treatment) or the outcome measure [32–34]. To support reproducibility, all scientific data and metadata will be made available following completion of the study. Thus, the development, design, and conduct of the study will maximize the potential for the study to fill critical gaps in evidence and the findings to be adopted into clinical practice [14].

Although the CISTO Study will address important evidence gaps in the management of recurrent high-grade NMIBC, there are limitations to the study design. Most notably, participants in the CISTO Study are not randomized to a treatment arm but instead select their treatment. However, similar randomized controlled trials comparing radical surgery with more conservative management strategies for genitourinary malignancies suffered from substantial accrual challenges [38, 39]. BRAVO was a randomized trial to establish the feasibility of comparing intravesical BCG with radical cystectomy in high-risk NMIBC. BRAVO demonstrated that patients had low willingness to be assigned to a treatment, halting continuation of a future randomized trial [9]. Therefore, a prospective observational cohort study was selected as the highest quality design for the CISTO Study to address the research questions. An additional limitation is the need for continued follow-up beyond the primary endpoint of 12 months to assess the long-term comparative effectiveness of BST vs. radical cystectomy.

Despite these limitations, the CISTO Study is the only ongoing clinical trial evaluating radical cystectomy as a comparator in NMIBC. A recent Clinical Trial Planning Meeting of the National Cancer Institute’s Genitourinary Cancers Steering Committee Bladder Cancer Task Force entitled “Defining the Next Generation of Clinical Trials with Combination Therapies in Non-Muscle Invasive Bladder Cancer” reviewed concepts for next generation trials in NMIBC, none of which included radical cystectomy as a comparator intervention [40]. Yet guidelines continue to advocate consideration of radical cystectomy for recurrent high-grade NMIBC, [3] making the results of the CISTO Study critical for continuing to evaluate the role of cystectomy in recurrent bladder cancer care.

The CISTO Study will advance clinical care by generating new and actionable evidence to support the decision-making of patients with recurrent high-grade NMIBC considering BST or radical cystectomy, including PRO and clinical outcomes. The results from this study will have direct patient care implications as bladder cancer is among the most common cancers in the United States.^1^ Future work will focus on the dissemination and implementation of findings through risk prediction models that integrate PRO and clinical outcomes data to permit the identification of which patients benefit from radical cystectomy and which patients benefit from BST. The CISTO study will provide an evidence-based approach to identifying the right treatment for the right patient at the right time in the challenging clinical setting of recurrent high-grade NMIBC.

### Supplementary Information

**Additional file 1: Supplemental Figure 1**. CISTO Study sites. A map of the 36 sites participating in the CISTO Study. **Supplemental Table 1.** Candidate baseline [41-43].

## Data Availability

Data sharing is not applicable to this article as no datasets were generated or analyzed for the current study. In accordance with the PCORI Policy for Data Management and Data Sharing, all data and metadata generated from the CISTO Study will be deposited in a PCORI-designated data repository within 1 year after completion of the study and available for third-party requests.

## Abbreviations

AE: Adverse event
AHRQ: Agency for Healthcare Research and Quality
ASA: American Society of Anesthesiologists
BCAN: Bladder Cancer Advocacy Network
BCG: Bacillus Calmette-Guérin
BCI: Bladder Cancer Index
BST: Bladder-sparing therapy
CISTO: Comparison of Intravesical Therapy and Surgery as Treatment Options
COST: Comprehensive Score for Financial Toxicity
CTCAE: Common Terminology Criteria for Adverse Events
ECOG: Eastern Cooperative Oncology Group
ED: Emergency Department
EHR: Electronic health record
EORTC QLQ-C30: European Organisation for Research and Treatment of Cancer Core Quality of Life
GEE: Generalized estimating equations
IRB: Institutional Review Board
ITT: Intention-to-treat
NMIBC: Non-muscle invasive bladder cancer
PCORI: Patient-Centered Outcomes Research Institute
PRECIS-2: PRagmatic-Explanatory Continuum Indicatory Summary 2
PRO: Patient-reported outcome
PROMIS: Patient Reported Outcome Measurement Information System
QOL: Quality of life
RUCA: Rural–Urban Commuting Area
SAE: Serious adverse event
SDH: Social determinants of health
SILS: Single Item Literacy Screener
SPIRIT: Standard Protocol Items: Recommendations for Interventional Trials
TMLE: Targeted maximum likelihood estimation
TTO: Time tradeoff
