# Supplementary material for: Protocol of the Comparison of Intravesical Therapy and Surgery as Treatment Options (CISTO) study: a pragmatic, prospective multicenter observational cohort study of recurrent high-grade non-muscle invasive bladder cancer
Source: BMC Cancer. 2023 Nov 18;23:1127. doi: 10.1186/s12885-023-11605-8 (PMC10657633; doi:10.1186/s12885-023-11605-8)
Supplement: Supplementary file 1 — Additional file 1: Supplemental Figure 1. CISTO Study sites. A map of the 36 sites participating in the CISTO Study. Supplemental Table 1. Candidate baseline [41-43]. [file 12885_2023_11605_MOESM1_ESM.docx]

**Supplementary materials for “Protocol of the Comparison of Intravesical Therapy and Surgery as Treatment Options (CISTO) Study: a pragmatic, prospective multicenter observational cohort study of recurrent high-grade non-muscle invasive bladder cancer”**

1. Supplementary Figure 1.

2. Supplementary Table 1.

**Supplemental Figure 1. CISTO Study sites.** A map of the 36 sites participating in the CISTO Study.


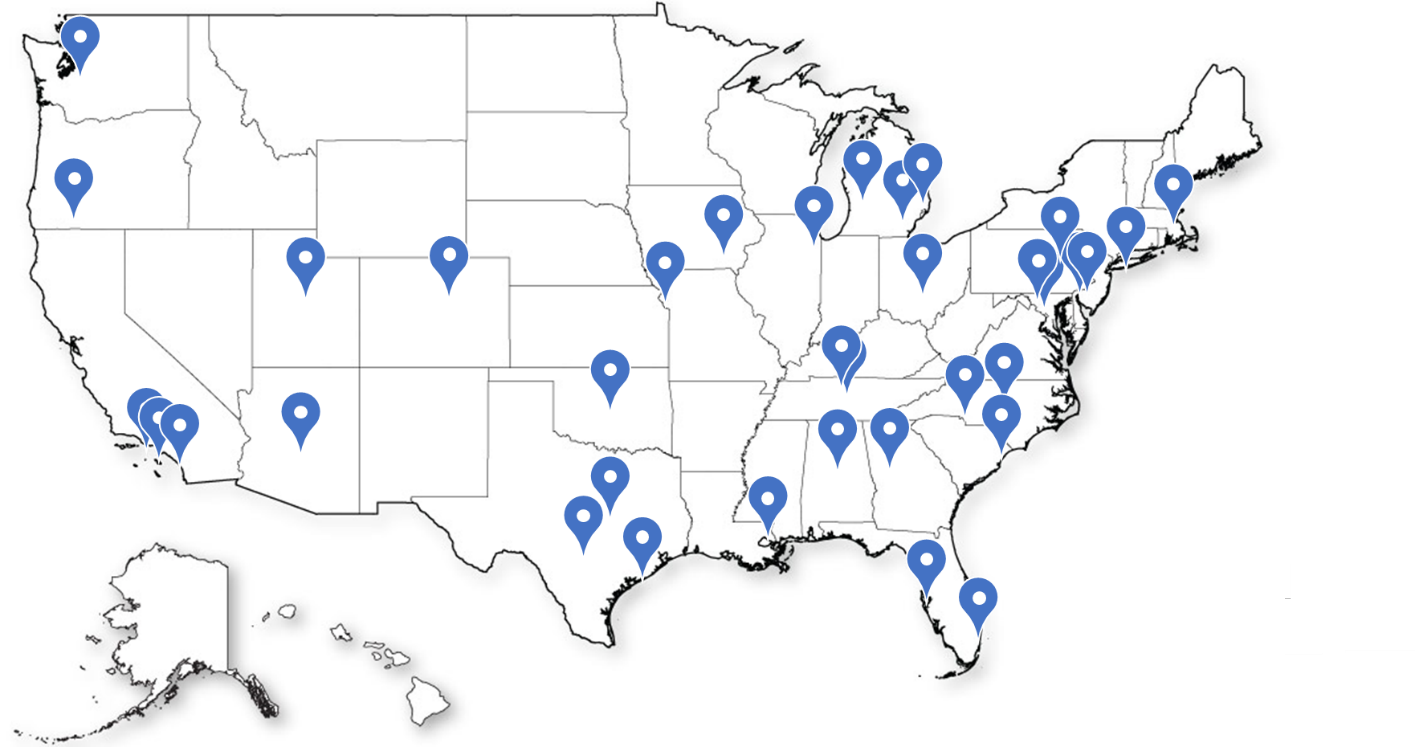


**Supplemental Table 1. Candidate baseline variables to be included in TMLE models.**

| **Variable** | **Variable Specification** |
| --- | --- |
| Age | Age at enrollment as continuous and categorical variable |
| Gender | Male, female, other |
| Race/ethnicity | Non-Hispanic White vs. all other racial/ethnic groupings |
| Comorbid health conditions | Enumerated comorbidities; ECOG performance status; PROMIS Emotional Distress-Anxiety/Depression – Short Forms 4a |
| Smoking history | Pack-years; categorized as active, former, never |
| Cancer severity  Stage  Cancer histology  Multifocality  Size | Tumor stage classification (T2 or T3)  Urothelial vs. mixed histology with atypical variant  Unifocal or multifocal tumor  Largest diameter (cm) |
| Caregiver status | Partnered vs. unpartnered |
| Social determinants of health | Area Deprivation Index based on ZIP Code(41); Employment Status; Combined Family Income; Education Level;  Health Care / Prescription Drug Coverage |
| Rurality | Rural-Urban Commuting Area (RUCA) code based on ZIP code(42) |
| Patient preferences | Preferences for health states relevant to NMIBC |
| Health literacy | Single Item Literacy Screener (SILS)(43) |
| Baseline QOL | Baseline EORTC QLQ-C30(22) and BCI(25) |

ECOG: Eastern Cooperative Oncology Group

TMLE: targeted maximum likelihood estimation; ECOG: Eastern Cooperative Oncology Group; PROMIS: Patient Reported Outcome Measurement Information System, NMIBC: non-muscle-invasive bladder cancer; QOL: quality of life; EORTC QLQ-C30: European Organisation for Research and Treatment of Cancer Core Quality of Life; BCI: Bladder Cancer Index.
